# Flame Retardancy and Thermal Stability of Rigid Polyurethane Foams Filled with Walnut Shells and Mineral Fillers

**DOI:** 10.3390/ma17184629

**Published:** 2024-09-21

**Authors:** Sylwia Makowska, Karolina Miedzińska, Agnė Kairytė, Jurga Šeputytė-Jucikė, Krzysztof Strzelec

**Affiliations:** 1 Institute of Polymer & Dye Technology, Lodz University of Technology, 90-924 Lodz, Poland or sylwia.makowska@p.lodz.pl (S.M.); karolina.miedzinska@dokt.p.lodz.pl (K.M.); krzysztof.strzelec@p.lodz.pl (K.S.); 2 Civil Engineering Research Centre, Vilnius Gediminas Technical University, Saulėtekio Av. 11, 10223 Vilnius, Lithuania; 3 Laboratory of Thermal Insulating Materials and Acoustics, Faculty of Civil Engineering, Institute of Building Materials, Vilnius Gediminas Technical University, Linkmenu St. 28, LT-08217 Vilnius, Lithuania; jurga.seputyte-jucike@vilniustech.lt

**Keywords:** polyurethane, walnut shells, perlite, vermiculite, thermal stability, fire resistance

## Abstract

Recently, the influence of the concept of environmental sustainability has increased, which includes environmentally friendly measures related to reducing the consumption of petrochemical fuels and converting post-production feedstocks into raw materials for the synthesis of polymeric materials, the addition of which would improve the performance of the final product. In this regard, the development of bio-based polyurethane foams can be carried out by, among other things, modifying polyurethane foams with vegetable or waste fillers. This paper investigates the possibility of using walnut shells (WS) and the mineral fillers vermiculite (V) and perlite (P) as a flame retardant to increase fire safety and thermal stability at higher temperatures. The effects of the fillers in amounts of 10 wt.% on selected properties of the polyurethane composites, such as rheological properties (dynamic viscosity and processing times), mechanical properties (compressive strength, flexural strength, and hardness), insulating properties (thermal conductivity), and flame retardant properties (e.g., ignition time, limiting oxygen index, and peak heat release) were investigated. It has been shown that polyurethane foams containing fillers have better performance properties compared to unmodified polyurethane foams.

## 1. Introduction

In Europe, buildings consume 40% of the total energy that is used for space heating and cooling. Improving the insulation of buildings would contribute to reducing CO_2_ emissions and energy costs. Decreasing heat transfer via advanced thermal insulators and making their production more sustainable are efficient approaches to improving energy efficiency and reducing the world’s total energy consumption, which contributes to a lower carbon footprint [1]. It is therefore important to develop new insulating materials with low thermal conductivity value (λ) to compete with commercially available insulating materials such as polystyrene, mineral wool, glass fiber, and bio-insulation materials [2]. Among polymeric materials, rigid polyurethane (PUR) foams represent a traditional material with a porous structure, which is commonly used as a thermally insulating material due to its low density and low thermal conductivity.

PUR is synthesized by the polyaddition reaction of aromatic or aliphatic diisocyanates with compounds containing at least two hydroxyl groups. The worldwide consumption of PUR was estimated at USD 60.5 billion in 2017, and it is predicted to be over USD 79 billion by 2023 [3,4]. Due to the versatility of their structures and properties, PUR is used for a variety of applications, including flexible and rigid foams—which account for more than 60% of the market—as well as coatings, adhesives, surfactants, and thermoplastic elastomers [5]. Rigid PUR foams are characterized by the best thermal insulation properties among the currently available polymeric materials. The value of the thermal conductivity of these materials ranges from 0.018 to 0.025 W·m^−1^·K^−1^ and is significantly lower than the values of this parameter for other known thermal insulation materials, such as expanded polystyrene or mineral wool [6]. Due to this, rigid PUR foams find varied applications across several areas, e.g., in construction, the automotive industry, industrial insulation (room insulation, transport vehicles, and piping), and household appliances [7,8].

Recently, there has been an increase in the impact of the concept of sustainable development, which includes environmental activities connected with reducing the use of petrochemical-derived fuels and converting post-production feedstocks into raw materials for the synthesis of polymeric materials [9,10]. In 2019, the global PUR market based mainly on petrochemical feedstock was valued at USD 95.13 billion and is expected to reach USD 149.91 billion by 2023 [8]. As a result, intensive efforts are being made to reduce the use of fossil fuels and replace them with environmentally friendly solutions. In this regard, the development of bio-based PUR foams can be performed by the modification of PUR foams with the addition of plant or waste fillers [6]. Previous studies have shown that the introduction of bio-based fillers into PUR foams improves their physical and mechanical properties, as well as provides reactive groups that can react with isocyanates. A variety of fillers derived from agricultural and forestry wastes have been studied in the literature.

For example, Jabber et al. [11] added 1, 3, and 5 wt.% of cellulose fibers from pineapple to polyurethane foams. As the fiber content increased, the apparent density and compressive strength decreased, but the authors highlighted that composite foams still exhibited values that met standards for building insulation applications. Cellulose was used as a filler for rigid polyurethane foams also by Uram et al. [12]. The system contained 40 wt.% canola oil-based polyol, while filler was used in amounts of 1, 2, and 3 wt.%. It was found that the addition of the microcellulose filler influenced a decrease in cell size and an increase in the close cell content. In addition, the foams were dimensionally stable, and their compressive strength in the direction parallel to the foam growth exceeded 200 kPa. Khaleel et al. [13] used turkey feather fibers at 0–15 wt.% as an additive in polyurethane foams. Their study showed that the incorporation of up to 6 wt.% of fibers improved the thermal stability of the composites, while a higher amount lost this effect. The lowest thermal conductivity was exhibited by the sample containing 3 wt.% of filler. SEM images showed that foams with low levels of filler showed uniform structure, while an increase in the amount of modifier resulted in a distorted cellular structure, which also had consequences in the air permeability test. Based on the obtained results, the authors concluded that the optimal level of turkey feather fibers was 3 wt.%. Wrześniewska-Tosik et al. [14] obtained semi-rigid polyurethane foams with the addition of 10 wt.% of keratin flour from poultry feathers and various flame-retardant additives. Their research has shown that the use of keratin filler significantly reduces the amount of smoke generated during combustion. In another study, Wrześniewska-Tosik et al. [15] used milled poultry feathers as an additive for elastic polyurethane foams. Their examination showed that the modified foams exhibited an extended ignition time, higher limiting oxygen index values, and a lower combustion temperature. The addition of keratin resulted in a reduction in the density of the foams and a deterioration of the sound absorption capacity.

Despite the use of many biofillers, new lignocellulosic fillers are still being investigated, which would be able to successfully improve the properties of the insulating materials obtained and, in addition, would be a waste product for which a sustainable disposal option could be found. An example of a filler that meets the requirements is walnut shells. Walnut (*Juglans regia* L.) is a crop grown in many regions due to its edible nuts [16]. The worldwide production of walnuts is increasing continuously, reaching 2.31 million metric tons in 2021–2022, and is expected to reach 2.6 metric tons in 2022–2023 [17]. The walnut fruit mainly consists of green husk, shell, skin, and edible kernel. As walnut shells constitute up to 67% of the total weight, there are more than 1.5 million tons of walnut shells left over worldwide each year [18]. The disposal of walnut shells as a by-product of agriculture has become a kind of environmental problem. They are usually thrown away or burned. However, they are difficult to decompose, and their combustion generates significant amounts of carbon dioxide [19,20]. Walnut shells consist mainly of lignin 50%, cellulose 25%, and hemicelluloses 22% [21,22,23]. They also contain smaller quantities of proteins, fat, waxes, and essential oils. Their high availability, low cost, high strength, and environmental friendliness have made them an interesting material for use in construction and composite materials [17].

Considering the main disadvantage of PUR foams is their high flammability, the present study focuses on the physical functionalization of walnut shells with natural flame retardant compounds—vermiculite (V) and perlite (P)—using a high-energy ball milling process. Given the beneficial properties of walnut shells and natural flame retardants (vermiculite and perlite), it is anticipated that the PUR composites developed in this study will exhibit exceptional mechanical and thermal properties, extending their use in construction. Consequently, the impact of the developed biofillers on the mechanical, thermal, insulation, and performance properties of PUR composites will be investigated.

## 2. Materials and Methods

### 2.1. Materials

- Polyol—*Stapanpol PS-2352* (polyester polyol)—Stepan Company (Northfield, IL USA);
- Isocyanate—*Purocyn B* (4,4′-diphenyl-methane-diisocyanate)—Purinova (Bydgoszcz, Poland);
- Surfactant—*Tegostab B8513* (silicone surfactant)—Evonik (Essen, Germany);
- Catalysts—*Kosmos 75* (Potassium octoate), Kosmos 33 (Potassium acetate)—Evonik (Essen, Germany);
- Foaming agent—pentane, cyclopentane (physical blowing agent)—Sigma-Aldrich Corporation (Saint Louis, MO, USA), water (chemical blowing agent);
- Walnut shells—Local Company (Lodz, Poland);
- Vermiculite—FlameHunter VE MIC (Mg,Fe,Al)_3_(Al,Si)_4_O_10_(OH)_2_·4H_2_O)—NYSA CHEM (Wrocław, Poland);
- Perlite—Sigma-Aldrich (Saint Louis, MO, USA).

### 2.2. Synthesis of Polyurethane Foams

**Figure 1:**
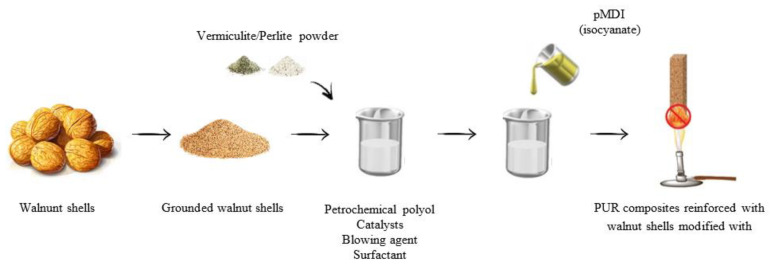
Scheme for the synthesis of polyurethane foams.

The synthesis of PUR foams was carried out by the one-shot method. In the first step, the calculated amounts of polyol, surfactant, catalysts, and blowing agents were placed in a container and mixed with a mechanical stirrer at 1000 RPM for 30 s. In the next step, the isocyanate was added to the container and mixed at 1000 RPM for 30 s until the ingredients were evenly combined (Figure 1). The compositions of the obtained polyurethane foams are presented in Table 1 and Table 2, where PU_0 means reference foam, PU_X means foam with the addition of a filler or a combination of nutshell/mineral fillers, and the “-” sign means that no filler was used.

The obtained mixture was left in the container, allowing free growth in the vertical direction. In the case of individual walnut shells, 10 parts by weight based on polyol were used. However, in the case of combinations of nutshell/mineral fillers, their mutual ratios (3:1, 1:1, and 1:3) were used, which, in total, also amounted to 10 parts by weight relative to the polyol.

### 2.3. Methods

The grinding of the nutshells to powder was carried out using the Monsieur Cuisine SKMC 1200 E5 (Lidl Stiftung & Co. KG, Neckarsulm, Germany). The grinding was carried out in 5 series lasting 2 min with the rotations set to the maximum speed on a 10-degree scale. After grinding, the obtained fillers were dried in a dryer for 2 h at 30 °C. To optimize and simplify the process, the fillers after grinding were not sieved or separated into fractions.

The particle size of the fillers was measured using the Zetasizer NANOS90 apparatus (Malvern Panalytical, Westborough, MA, USA). The mass concentration of filler particles in the polyol was 0.04 g/L. The measurement was repeated at least five times for each sample and the results are presented as an average.

The surface of fillers and composite foam morphology were analyzed using an optical microscope LAB 40 M from OPTA-TECH (Warsaw, Poland). To conduct a more accurate analysis, images were taken at various magnifications (50× and 100×). Microscopic images of the structures of the obtained PU foams were analyzed using computer software (ImageJ v.153i). The anisotropy factor was defined as the ratio of the length to the width of the cell. The cell structure parameters of PU foams were determined from at least four microscopic images of different samples. The photos shown in the experimental section are selected overview photos.

The fillers and composite foams were examined by using Fourier-transform infrared spectroscopy (FTIR) and near-infrared spectroscopy (NIR), which allows for analysis of the functional groups that are present in materials. The measurement was conducted using a ThermoScientific Nicolet 6700 spectrometer (Waltham, MA, USA) equipped with a diamond Smart Orbit ATR tool (FT-IR analysis), and a Smart NIR Integrating Sphere Accessory detector (NIR analysis). Fourier-transform infrared spectroscopy spectra were recorded within the 4000–400 cm^−1^ range (number of scans—128, resolution of 4 cm^−1^), and near-infrared spectroscopy spectra were recorded within the 10,000–4000 cm^−1^ range (number of scans—128, resolution of 4 cm^−1^). All the spectra were recorded to the background spectra. The obtained spectra were analyzed using the OMNIC 9.2.86 software.

The dynamic viscosity of polyol systems was determined using a Brookfield DV-II+ (Middleborough, MA, USA) viscometer, following the ISO 2555 standard [24]. Before the test, the device was calibrated using the adjustment ring. The analyzed liquid with a volume of about 1 cm^3^ was placed in a sample cup, which was then mounted in the apparatus and secured with a special clamping lever. The viscosity measurements were made at 23 °C at the following shear rates: 10, 20, 50, and 100 rotates per minute. The measurement was repeated at least five times for each sample and the results are presented as an average.

The apparent density of polyurethane foams was assessed as the ratio of sample weight to its volume, following the ISO 845 [25]. The measurement was repeated at least five times for each sample and the results are presented as an average.

The hardness of polyurethane foams was determined using the Shore method, by the ISO 868 standard [26] using a Shore hardness tester type 00 by Zwick/Roell, equipped with a ball indenter with a diameter of 1.2 mm. During the examination, at least fifteen measurements were made for each sample, consisting of inserting an indenter into the material, and the results are presented as an average.

The compressive strength of the polyurethane foams was determined by the ISO 844 [27] using the Zwick Z100 Testing Machine (Zwick/Roell Group, Ulm, Germany) with a load cell of 2 kN. The compressive strength as a ratio of the load causing 10% cross-sectional strain of the samples was measured parallel to the foam growth direction, at least four times for each sample, and the results are presented as an average.

The flexural strength of polyurethane foams was established by the ISO 178 standard [28]. The examination was carried out on samples with dimensions of 100 mm × 10 mm × 5 mm, which were bent with a speed of 2 mm min^−1^.using a Zwick Z100 Testing Machine (Zwick/Roell Group, Ulm, Germany). For each sample, at least five measurements were performed, and the results are presented as an average.

Water absorption of polyurethane foams was measured by the ISO 2896 standard [29]. The examination was carried out on samples with dimensions of 40 mm × 40 mm × 40 mm. Before the measurement, the tested samples were dried and weighed. Subsequently, probes were immersed in distilled water to a depth of 10 mm. After 24 h, the samples were removed from water, dried with dry filter paper, and weighed. The measurement was completed at least four times for each sample and the result is presented as an average.

The surface hydrophobicity of polyurethane foams was measured by contact angle examination using the sessile drop method. The examination was conducted on samples with dimensions of 100 mm × 10 mm × 5 mm, using a manual contact angle OCA 15EC goniometer from DataPhysics Instruments GmbH (Filderstadt, Germany). The measurement consisted of depositing a drop of water with a volume of 1 μL, using a micrometer syringe, on the surface of the sample cut out of the inside of the foam and measuring the angle at the contact point of three phases: solid, liquid, and gas. Contact angle values were measured at least fifteen times for each sample, and the result is presented as an average.

The dimensional stability of polyurethane foams was determined by the ISO 2796 standard [30]. The dimensional stability was determined on samples with dimensions of 40 mm × 40 mm × 40 mm, based on the linear changes in dimensions and volume changes of polyurethane foams. The foams were exposed to elevated (+70 °C) and reduced (–20 °C) for 72 h. Dimensional stability values were measured at least fifteen times for each sample, and the result is presented as an average.

The thermal stability of polyurethane foams was assessed using a Mettler Toledo Thermogravimetric Analyzer TGA/DSC1 (Mettler Toledo, Greinfensee, Switzerland). The analysis included an examination of the mass change as a function of temperature. The thermal decomposition was conducted in the temperature range between 25 and 800 °C (heating rate of 10 °C min^−1^) and in an inert gas atmosphere (flow 50 mL min^−1^). The initial temperatures of subsequent stages of thermal decomposition were determined based on the inflection points in the DTG plot.

The burning behavior of polyurethane foams was determined by the ISO 5660 standard [31]. During the analysis, the following flammability properties were assessed: ignition time IT, total heat release THR, total smoke release TSR, and the maximum average rate of heat emission (MARHE). The examination was conducted on samples with dimensions of 100 mm × 100 mm × 50 mm, using a cone calorimeter in S.Z.T.K. TAPS (Maciej Kowalski Company, Lodz, Poland). Every sample was wrapped in aluminum foil and burned at an external heat flux of 35 kW m^−2^. The examination was performed two times for each sample, and the results are presented as an average. The limited oxygen index (LOI) was measured using NETZSCH TAURUS Co., Ltd., in Weimar, Germany, in accordance with ISO 4589 [32]. .

The UL94 test was performed by the ASTM D3801-10. The examination was conducted on samples with dimensions of 130 mm × 13 mm × 10 mm, using a UL94 Horizontal/Vertical Flame Chamber (FTT, East Grinstead, UK). The examination was carried out five times for each sample, and the results are presented as an average.

The thermal conductivity of polyurethane foams was assessed by the ISO 8301 standard [33], using HFM 446 Lambda Series (NETZSCH, Selb, Germany). The examination was carried out on samples with dimensions of 200 mm × 200 mm × 20 mm. Thermal conductivity was assessed at mean temperatures of 10, 20, and 40 °C with a load of 2 kPa. The temperature presented in the analysis was the average value, while the temperatures of the plates on the two sides of the analyzed foam differed by 11 °C at each stage and were 4.5, 14.5, and 34.5 °C for the lower plate, and 15.5, 25.5, and 45.5 °C for the upper plate, respectively. Thermal conductivity values were measured at least three times for each sample, and the result is presented as an average.

## 3. Results and Discussion

### 3.1. Filler Characterization

#### 3.1.1. Optical Microscopy

**Figure 2:**
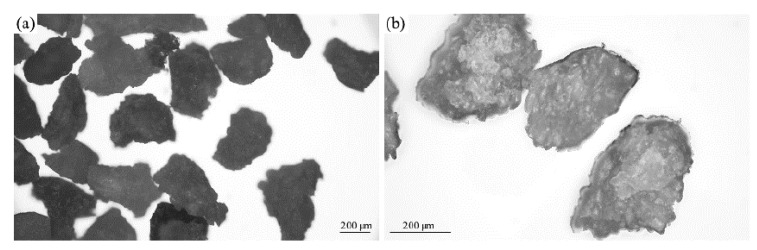
Optical image of walnut shells at magnification of (**a**) 50 and (**b**) 100.

The surface structure of mineral fillers and walnut shell fillers was examined using optical microscopy. Figure 2a,b shows images of walnut shells obtained at 50× and 100× magnifications. Based on these images, it can be noticed that the walnut shells showed similar sizes and irregular shapes. Their surface appears to be rough, which may be due to the natural structure of these shells. In addition, differences in light reflections may indicate the presence of a layer of wax on their surface, which is their natural component.

**Figure 3:**
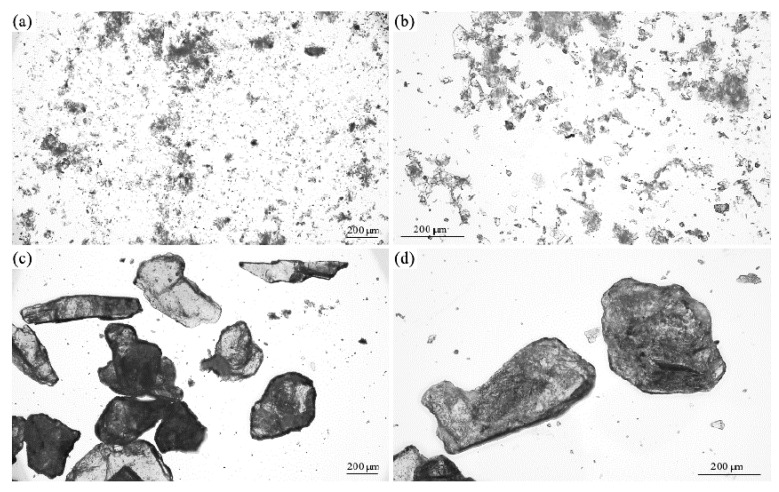
Optical images of (**a**,**b**) perlite, and (**c**,**d**) vermiculite at 50× (**a**,**c**,) and 100× (**b**,**d**,) magnification.

The surface structure of mineral fillers is presented in Figure 3a–d. Based on these images, it can be noticed that they showed a diverse structure. The perlite particles (Figure 3a,b) were noticeably the smallest and most diverse. They take the form of small spherical, irregular particles, but you can also observe some particles resembling flat crystals. Vermiculite was characterized by large particles, resembling crystals with irregular shapes (Figure 3c,d). Their surface appeared relatively smooth with visible scratches and areas as if the stone had been split.

#### 3.1.2. Size Distribution

**Figure 4:**
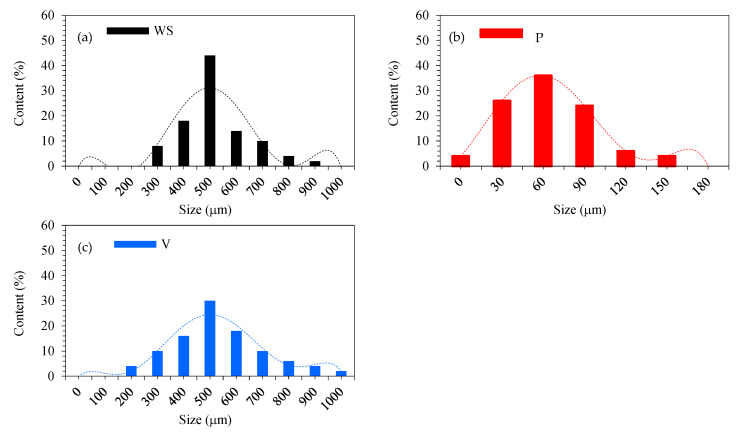
Size distribution of fillers: (**a**) walnut shells; (**b**) vermiculite; and (**c**) perlite.

According to the results of the dynamic light scattering (DLS) method, presented in Figure 4a–c, the walnut shell particles are in a range of 200–1000 μm; however, about 76% of their particles occurred in the range between 400 and 600 μm with a peak of 44% at ~500 μm. The broadest (200–1000 μm) particle size range was exhibited by vermiculite.

The 400–600 μm range contained about 64% of all particles, while the largest share of 30% was observed for the ~500 μm. By far the smallest particle size among all the fillers showed perlite, most of whose particles did not even exceed 100 μm. In the case of perlite, the largest share of 36% was reached by particles in the size of ~60 μm, while as many as 86% of the particles were in the range of 30–90 μm.

#### 3.1.3. FTIR/NIR Characterization

**Figure 5:**
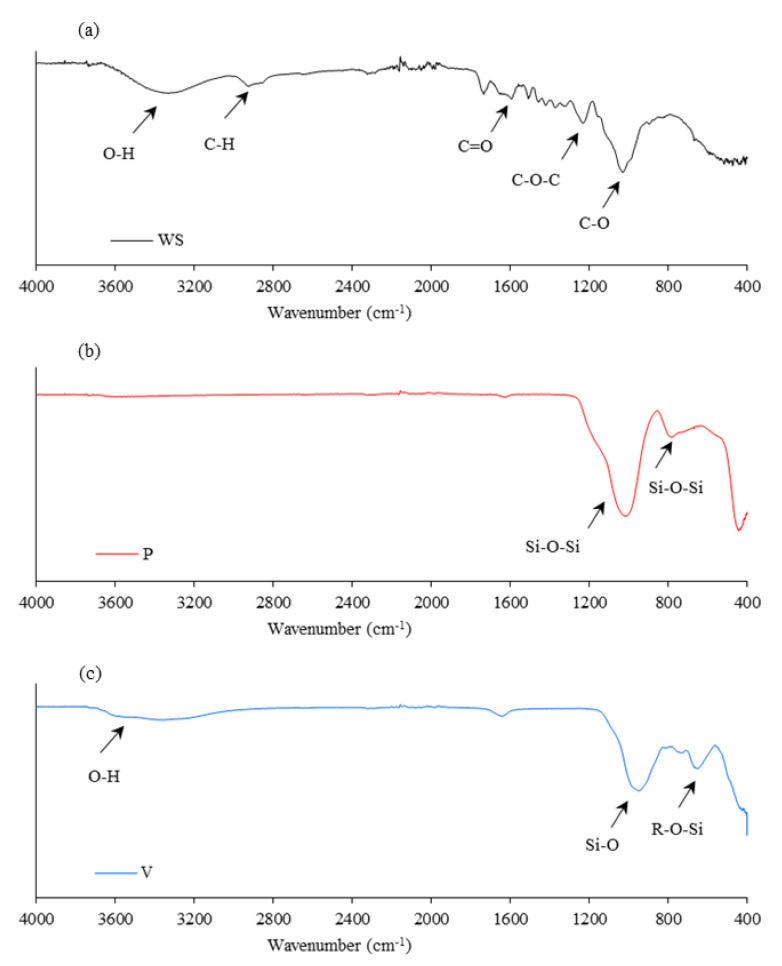
FTIR spectra of (**a**) walnut shells, (**b**) perlite, and (**c**) vermiculite.

Figure 5a shows the FTIR spectra of walnut shells. Analyzing the FTIR spectrum, the broad peak at 3300 cm^−1^ corresponds to the O–H stretching oscillation of the hydroxyl group, and the beaks between 2980 and 2810 cm^−1^ are the C–H stretching vibrations. The peaks at 1730 cm^−1^ and 1600 cm^−1^ are related to the stretching C=O vibrations of the carbonyl group of hemicelluloses. The peak at wavelength of 1230 cm^−1^ may be associated with C–O–C stretching, while the strong peak at 1030 cm^−1^ corresponds to C–O stretching. The last quite broad peak may be related to C–C and C–H stretching [34,35,36]. Analyzing the FTIR spectrum, a broad peak with a maximum at the range of 8200–8100 cm^−1^ is assigned to the C–H stretching vibration of cellulose. The region between 7500 and 6000 cm^−1^ corresponds to the overlap of signals of various types of cellulose –OH groups. The peak between 5200 and 5100 cm^−1^ is related to the –OH vibrations of water, and the peak at 4740 cm^−1^ is assigned to the O–H stretching oscillation of cellulose [37,38,39].

Analyzing the FTIR spectrum of perlite (Figure 5b), the peak at 1015 cm^−1^ may be assigned to Si–O–Si asymmetric stretching vibrations, the band at 785 cm^−1^ is related to the bending motion of the Si–O–Si oxygen atom along the bisector of the bridge, and the peak at 440 cm^−1^ is due to the rocking motion of the Si–O–Si oxygen atom perpendicular to the plane [40,41,42]. When analyzing the FTIR spectrum of vermiculite (Figure 5c , the region between 3800 and 3000 cm^−1^ may be assigned to the O–H stretching vibration of adsorbed water molecules.

The weak band at 1640 cm^−1^ is related to the –OH bending oscillation of adsorbed water. The peak at 950 cm^−1^ may be related to the Si–O stretching oscillations, and the band at 645 cm^−1^ can be attributed to R–O–Si (where R = Al, Mg, Fe, etc.) oscillations of tetrahedral sheets. The last two peaks are slightly offset from those reported in the literature (~1000 and ~680 cm^−1^), but the overall shape of the spectrum is analogous [43,44,45].

#### 3.1.4. Thermogravimetry Analysis

**Figure 6:**
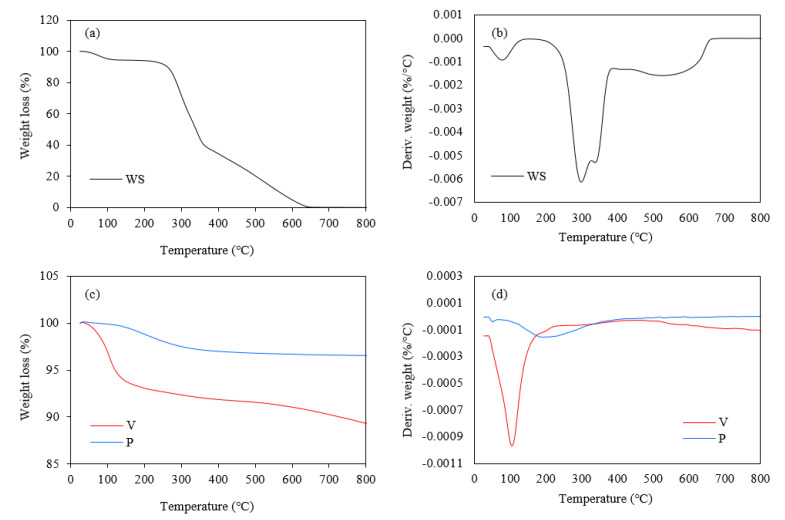
Thermogravimetric and derivative thermogravimetric results of walnut shells (**a**,**b**), vermiculite, and perlite (**c**,**d**).

To evaluate the thermal stability of the walnut shells, the thermogravimetric analysis (TGA) and derivative thermogravimetric analysis (DTG) were performed. During the examination, the subsequent decomposition stages were determined from the inflection points on the DTG curve, as well as the char residues at 600 °C. The obtained results of the thermal analysis are presented in Figure 6a,b and summarized in Table 3.

Walnut shells, like other lignocellulosic materials, are mainly composed of cellulose, hemicelluloses, and lignin [46,47,48,49]. The thermogravimetric behavior of these components has been analyzed and it is known that the decomposition of hemicelluloses occurs in the temperature range of 210–325 °C, cellulose in the temperature range of 310–400 °C, while the lignin decomposition occurs over a wide temperature range covering 160–900 °C [36,50].

Based on the data, it can be concluded that walnut shells showed three main stages of thermal decomposition. The first stage, which for all fillers occurred at about 77 °C, is usually associated with the evaporation of moisture and volatile components present in the samples.

The second stage is related to the decomposition of organic components such as hemicelluloses and cellulose [51,52,53]. For walnut shells, it occurred at 296 °C. In excess, additional intermediate peaks associated with this stage can also be observed at 338 °C. These may occur due to the differences in the temperature range of hemicelluloses and cellulose decomposition. It is considered that the lower temperature shoulder refers to the decomposition of hemicelluloses, while the higher value represents the decomposition of cellulose [54]. The third stage occurred at apparently the highest temperatures and was associated with lignin degradation [55,56]. This step occurred at 524 °C for walnut shells. Analysis of the char residues at 600 °C showed that the percentage of residue was 5.2%.

To assess the thermal stability of the mineral fillers, the thermogravimetric analysis and derivative thermogravimetric analysis were performed as well. During the analysis, the decomposition stages were evaluated from the inflection points on the DTG curve, as well as the residues at 800 °C. The results of the thermal analysis are presented in Figure 6c,d and summarized in Table 3. Due to the differences in the structure of the selected mineral fillers, they also exhibited different thermogravimetric specifications. Considering perlite, the maximum of the only slight peak determined from the DTG plot occurs at 193 °C and is most likely due to the removal of adsorbed surface water [57]. The continuation of a slight decrease in a sample weight at the higher temperature may be related to the volatilization of some trace metal oxides [57]. In the case of vermiculite, a single peak was also observed on the DTG plot at 104 °C. The mass loss that begins at this temperature is probably related to the evaporation of the surface and, as the temperature increases, the interlayer adsorbed water as well [45].

The residue analysis at 800 °C showed different contents for these fillers. Perlite had the highest residual content, which was 96.4%, and apparently showed the highest thermal stability. Vermiculite also showed a high residual content of 87.8%, indicating high thermal stability. For both of these fillers, the weight loss over the analyzed temperature range was most likely only due to the water evaporation, and no significant changes in structure occurred.

### 3.2. Properties of Foams with Walnut Shells and Mineral Fillers

#### 3.2.1. Synthesis of Polyurethane Foams

The synthesis of polyurethane foams containing mixtures of walnut shells and mineral fillers was carried out using a one-step method. After adding the isocyanate component to the polyol component with fillers, the mixture was thoroughly mixed and allowed to grow freely. The characteristic processing times (start, expansion, and stabilization) and maximum temperature were measured as presented in Table 4.

When analyzing the characteristic times of foams with combinations of walnut shells and mineral fillers, it can be observed that foams with fillers generally showed extended synthesis times compared to the reference foam. All the foams showed similar start times ranging from 29 to 31 s. As with single filler foams, the differences in characteristic times were more pronounced for foam expansion and stabilization times. Foams with added perlite showed the greatest differences in expansion time. Foams with combinations of fillers achieved times between those with single fillers PU_10WS (270 s) and PU_10P (287 s). These times increased with increasing perlite content, reaching 273, 279, and 284 s for PU_7.5WS_2.5P, PU_5WS_5P, and PU_2.5WS_7.5P foams, respectively. On the other hand, the stabilization times of the foams with walnut shells/perlite fillers were similar, reaching 379–380 s, and were similar to the PU_10WS foam. The total foam synthesis time was 642 s for the reference foam, 680 s for the PU_10WS foam, 682, 688, and 694 s for the walnut shells/perlite-filled foams, and 712 s for the PU_10P foam. Thus, it can be seen that the process length increased with increasing perlite content.

For the foams with walnut shells and vermiculite fillers, a similar relationship was observed for the expansion times as for the perlite foams. The expansion times of the foams increased from PU_10WS foam (270 s) to 277 s for PU_7.5WS_2.5V foam, 280 s for PU_5WS_5V and up to 285 s for PU_2.5WS_7.5V foam, which reached a longer time than PU_10V (281 s). The stabilization times of these foams also increased in the direction of the foams with higher vermiculite content. Similar relationships have been demonstrated in previous work [58,59]. The authors explained that this could be related to the increased viscosity of the filler-containing systems, resulting in a hindered expansion of gas bubbles and, consequently, a slower growth of the polyurethane mixture [58,59].

In terms of the maximum temperature of the process, the effect of the fillers was observed to increase the temperature compared to the reference foam, which reached 121.8 °C. For the foams with a perlite addition, an increase in the maximum process temperature was observed from 129.7 °C for PU_10WS and PU_7.5WS_2.5P foams, through 130.2, and 130.5 °C for PU_5WS_5P and PU_2.5WS_7.5P foams, to 131.4 °C for PU_10P foam. Thus, as the perlite content increased, the maximum foam synthesis temperature also increased. Foams containing vermiculite also showed higher maximum process temperatures with higher mineral filler content. The PU_7.5WS_2.5V and PU_5WS_5V foams showed temperatures of 129.9 and 132.0 °C, while the PU_2.5WS_7.5V foam showed a temperature of 134.4 °C, exceeding the PU_10V foam, which reached 134.1 °C. The higher temperature of polyurethane systems containing added fillers may be related to the presence of a small amount of water that is introduced into the system together with the filler. As a result of an exothermic reaction between the introduced water and the isocyanate groups, more heat is released, generating a higher system temperature during foam synthesis [60].

#### 3.2.2. Cellular Structure

**Figure 7:**
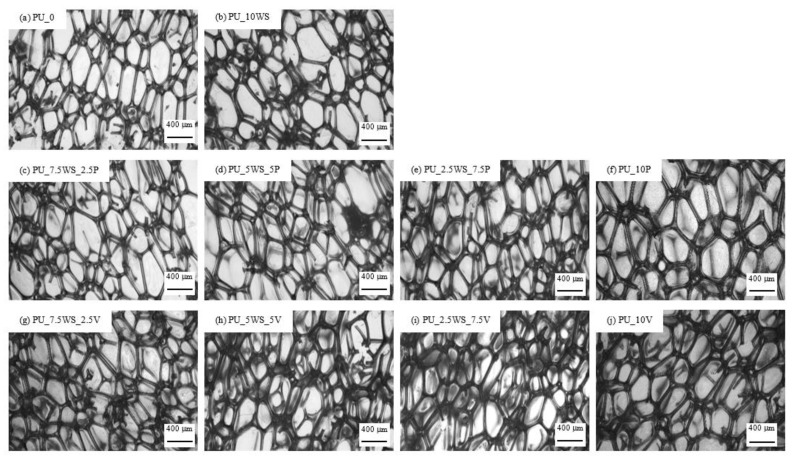
Morphology of polyurethane foams (**a**), polyurethane foams with walnut shells (**b**), polyurethane foams with the addition of walnut shells and perlite (**c**–**f**), polyurethane foams with the addition of walnut shells and vermiculite (**g**–**j**).

The cellular structure of polyurethane foams determines many important properties such as apparent density and mechanical and thermal properties [61,62]. The foams with the addition of combinations of walnut shells and mineral fillers were analyzed based on the microscopic images shown in Figure 7a–j, and the results obtained are summarized in Table 5.

From the microscopic images presented, it can be observed that the foams with the addition of walnut shells and mineral fillers display a typical polyhedral structure of cells separated by ribs. The cells undergo a notable elongation in the direction of foam growth, which is described by the anisotropy coefficient. Based on this parameter, it can be noticed that the foams with walnut shells/perlite combinations showed anisotropy coefficients (1.66–1.67) similar to PU_10WS foam (1.67), while foams with walnut shells/vermiculite combinations showed anisotropy coefficient values (1. 68–1.70) similar to PU_10WS (1.70). In general, all foams with walnut shells and mineral fillers showed a higher anisotropy coefficient compared to the reference foam (1.62), with a maximum difference of 5%.

Analysis of the data in Table 5 shows that all polyol systems with 10 parts of fillers (both single fillers and their combinations) had significantly increased dynamic viscosity at 10 RPM compared to the reference foam (780 mPa·s).

In general, polyol systems containing combinations of walnut shells and minerals showed intermediate viscosity values between the extremes reached by systems containing single fillers at 10 parts by weight, with an increasing or decreasing trend between them. The differences that have been observed in the viscosity values of polyol systems have also been reflected in the cellular structure of the foams and, consequently, in the apparent density values.

For foams with a walnut shells/perlite combinations addition, as viscosity increased toward higher perlite content, from 2820 mPa·s for PU_10WS to 3370 mPa·s for PU_10P, smaller and smaller average cell size (decrease from 498 to 411 μm), and, correspondingly, higher apparent density values were observed, from 38.17 kg m^−3^ through 38.23–42.03 kg m^−3^ for walnut shells/perlite foams. In the case of foams with walnut shells/vermiculite combinations, a completely different relationship can be observed—viscosity decreased with increasing vermiculite content (from 2820 mPa·s through 2810–2750 mPa·s, up to 2350 mPa·s), while, as in the previous case, the average cell size decreased (between 498 and 435 μm), and the apparent density values increased from 38.17 kg m^−3^ for PU_10WS through 38.22–39.18 kg m^−3^ for walnut shells/vermiculite foams, up to 39.54 kg m^−3^ for PU_10V. At this point, it is worth noting that the effect of fillers on the viscosity of polyol systems can be complex and may depend on the size of the filler particles, their shape and surface, or their properties. On the other hand, it can also be seen that a higher viscosity of a polyol system does not always result in a higher density of the final product.

From the data obtained, it can be seen that the addition of walnut shell filler, mineral fillers, and their combinations increased the dynamic viscosity of the polyol systems, reduced the average cell size, and increased the apparent density compared to the reference foam. The foams with added combinations of fillers also showed increased cell anisotropy. The foams showed a general trend of increasing density and decreasing cell size with increasing mineral filler content. In the case of dynamic viscosity, the addition of fillers certainly influenced the higher viscosity values achieved compared to the pure system, since all systems contained a total of 10 parts by weight of fillers and exhibited different dynamic viscosity levels, the specificity of the viscosity increase could be related to the properties of the fillers themselves, such as particle size, degree of surface development, their distribution, or tendency to aggregate.

#### 3.2.3. Mechanical Properties

**Figure 8:**
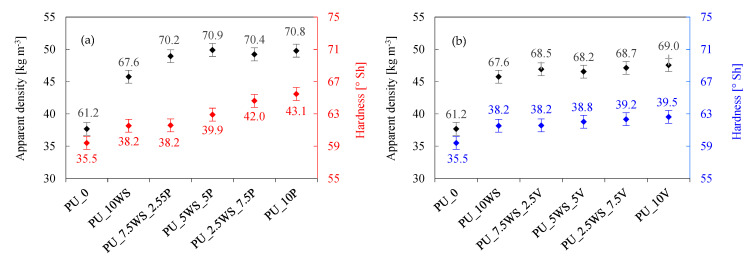
Effect of walnut shells and perlite (**a**) and walnut shells and vermiculite (**b**) on apparent density and hardness of polyurethane foams.

**Figure 9:**
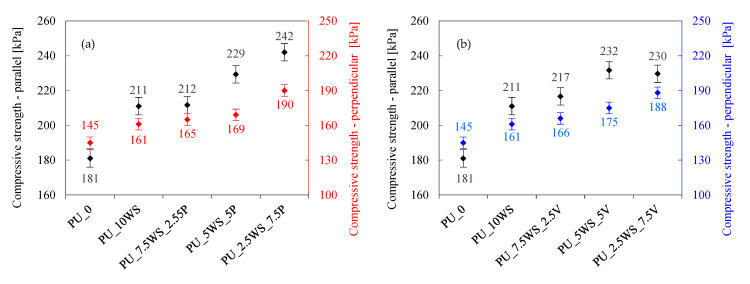
Effect of walnut shells and perlite (**a**) and walnut shells and vermiculite (**b**) on compressive strength of polyurethane foams.

**Figure 10:**
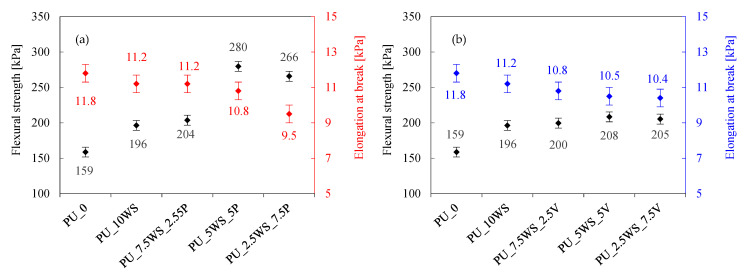
Effect of walnut shells and perlite (**a**) and walnut shells and vermiculite (**b**) on flexural strength and elongation of polyurethane foams.

The mechanical properties of foams are an important aspect in the context of their use in construction [61,62]. Depending on the application, foams should retain their structure and not deform under load. To evaluate the effect of the combination of natural and mineral fillers on the mechanical properties of polyurethane foams, hardness, compressive strength, and flexural strength tests were conducted, the results of which are shown in Figure 8, Figure 9 and Figure 10.

Analyzing the effect of the addition of combinations of walnut shells and mineral fillers on the hardness of polyurethane foams (Figure 8a,b), it can be observed that all the foams with the addition of fillers showed better results in terms of hardness than the reference foam (61.2 °Sh). All foams with added fillers achieved hardness values above 66.0 °Sh. The hardness results of the foams with a combination of fillers also showed higher values than the foam with walnut shells only, in addition, no clear trend is observed for the density of the foams. The highest values were obtained with foams containing perlite, which also showed the greatest improvement in this parameter compared to other mineral fillers. The highest score was achieved by PU_5WS_5P foam (70.9 °Sh), but it is worth noting that essentially all perlite foams achieved hardness scores in the range between 70.0 and 71.0 °Sh. In the case of foams with vermiculite addition, the highest score was achieved by the foam without walnut shell added, PU_10V (69.0 °Sh), and its closest result was represented by the foam PU_2.5WS_7.5PV with 68.7 °Sh.

Analyzing the effect of the addition of combinations of walnut shells and mineral fillers on compressive strength and flexural strength of polyurethane foams (Figure 9a,b and Figure 10a,b), it can be observed that all the foams with the addition of fillers showed higher compressive and flexural strength results when compared with the reference foam (compressive strength of 181 kPa and flexural strength of 159 kPa). In the case of foams with perlite, foams with the addition of the combination show similar or better mechanical results than foam with walnut shells alone; in addition, PU_5WS_5P and PU_2.5WS_7.5P foams also show better results than foam with perlite alone. The PU_5WS_5P foam achieved a compressive strength of 229 kPa and a flexural strength of 280 kPa, while the PU_2.5WS_7.5P foam achieved 242 and 266 kPa, respectively. In the case of foams with vermiculite-containing combinations, the flexural strength results of the foams were more similar to those with walnut shells alone (196 kPa), achieving results in the range of 200–208 kPa, while the foam with vermiculite alone achieved a significantly higher result of 257 kPa. All foams containing walnut shells/vermiculite combinations achieved higher compressive strength results than foams containing single fillers, in the range of 217–232 kPa (211 kPa in both cases).

As can be seen in the case of the mechanical properties of foams with a combination of walnut shells and mineral fillers, it is difficult to find an ideal trend or pattern between the compressive and flexural strength results. The compressive strength results show a similar trend to the increase in apparent density, where an increase in apparent density can explain the better compressive strength results by packing more material per unit volume and thus achieving better results. The results obtained in previous work [63] have confirmed that the type of filler added, as well as its size and shape, have a significant effect on the mechanical properties of the foams obtained.

#### 3.2.4. Water Uptake and Contact Angle

**Figure 11:**
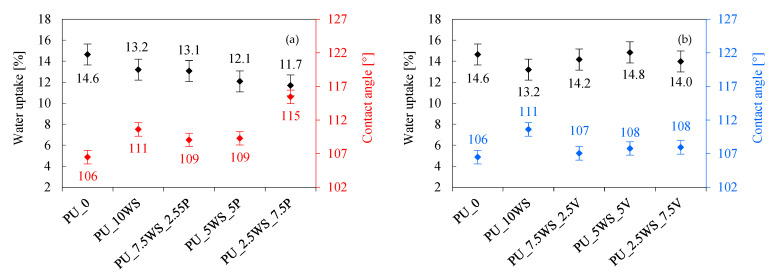
Water uptake and contact angle results of polyurethane foams with the addition of walnut shells and perlite (**a**), and walnut shells and vermiculite (**b**).

To determine the effect of adding combinations of natural and mineral fillers on water-related properties, water absorption and contact angle analyses of the obtained foams were performed, the results of which are presented in Figure 11a,b. When analyzing the results of polyurethane foams with the addition of walnut shells and mineral fillers, a general trend can be observed that as the mineral filler content increases, water absorption decreases, and contact angle values increase in parallel.

Furthermore, in the case of the series of foams containing walnut shells with perlite, the relationship is almost ideal, and the water absorption decreases from foams containing walnut shells only to foams containing mineral fillers. In this case, PU_10WS and PU_10V foams achieved water absorption of 13.2% and 12.9%, respectively, while foams containing combinations of these fillers showed higher water uptake results, ranging from 14.0% to 14.8%. In the case of walnut shells foams with perlite, the explanation for water absorption can be found in its dependence on density—a higher density combined with a higher proportion of mineral fillers reduces water absorption. For WS/V foams, it is difficult to determine exactly what is causing the increased water absorption, especially since the highest result is obtained with an equal proportion of walnut shells and vermiculite. Moreover, no analogous variation in foam density was observed to affect this behavior.

#### 3.2.5. Dimensional Stability

The dimensional stability analysis of the foams with the addition of combinations of natural and mineral fillers was completed under decreased (–20 °C) and increased (+70 °C) temperature conditions, and the results of linear changes in height (Δh), width (Δw), and thickness (Δt) are presented in Table 6. Analyzing foams with added walnut shells and mineral fillers, it can be observed that all foams, both at elevated and reduced temperatures, showed smaller dimensional changes than the reference foam. It is worth noting that at reduced temperatures, the foams performed better than at high temperatures.

#### 3.2.6. Thermogravimetry Analysis

**Figure 12:**
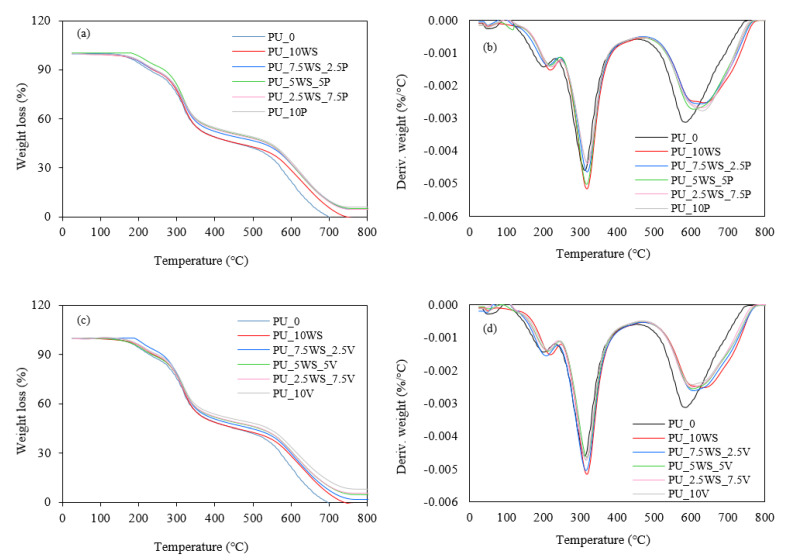
Thermogravimetric (TGA) and derivative thermogravimetry (DTG) results of polyurethane foams with the addition of walnut shells and perlite (**a**,**b**), and walnut shells and vermiculite (**c**,**d**).

To evaluate the effect of the addition of combinations of natural and mineral fillers on the thermal stability and the subsequent stages of thermal degradation of polyurethane foams, thermogravimetric analysis and derived thermogravimetric analysis were performed. The results of this study are presented in Table 7 and Figure 12a–d, whereas the TGA and DTG graphs have been omitted due to their similarity to previous chapters.

Analyzing the results of polyurethane foams with the addition of combinations of walnut shells and mineral fillers, it can be observed that, in general, the addition of all fillers increased the temperatures of subsequent thermal decomposition stages and the char residue at 600 °C compared to the reference foam. For foams with added perlite, the first stage is observed between 216 and 221 °C, the second at 319 °C, while the third stage shows the largest temperature differences between 608 °C (PU_5WS_5P) and 632 °C (PU_2.5WS_7.5P). The temperatures of the first two stages remained at the level of the foams with single fillers, but for the third stage, the foams with combinations achieved similar or lower temperature results. The char content at 600 °C increased with increasing perlite content from a value of 28.9% for PU_10WS foam, through 32.6–33.2% obtained for foams with the addition of the filler combinations, to the highest result of 34.2% obtained for PU_10P foam.

When analyzing the foams with vermiculite, it can be observed that the temperatures of the first stage of decomposition of foams with compositions were lower than those of foams with single fillers, reaching 207–212 °C. In the second stage, the temperatures of foams with fillers compositions were between 314 and 319 °C and were between those represented by foams with single fillers. For the third stage, the temperatures achieved between 604 and 608 °C were more similar to the temperature reached by PU_10V foam (604 °C). In the case of char residue at 600 °C, an increase in char content was observed with an increase in vermiculite content; for foams with a combination of fillers, the increase was between 30.3 and 31.7%.

When analyzing the foams with walnut shells and mineral fillers, it can be observed that all foams performed better than the reference foam, both in terms of higher temperatures of subsequent decomposition stages and higher char content at 600 °C. In general, the highest results were achieved by foams with the addition of perlite. For foams with the addition of perlite and vermiculite, the char content at 600 °C increased with increasing mineral filler content, reaching results above 30%.

#### 3.2.7. Burning Behavior

To assess the effect of the addition of the combinations of natural and mineral fillers on the flammability of polyurethane foams cone calorimetry tests were performed. Flammability tests were performed only on foams containing equal amounts of nutshells and mineral fillers. The ignition time (IT), total heat release (THR), total smoke release (TSR), carbon monoxide yield (COY), carbon dioxide yield (CO_2_Y), and maximum average rate of heat emission (MARHE) were determined during the study, and the results are presented in Table 8. When analyzing the burning behavior of polyurethane foams with the addition of walnut shells and mineral fillers, it can be observed that the ignition time increases from 4 s for the reference foam to 8 s for foams with the addition of walnut shells and perlite or vermiculite.

For the THR and TSR parameters, all foams with filler combinations show lower values than the reference foam (20.4 MJ m^−2^ and 750 m^2^ m^−2^), indicating less heat and smoke release during combustion. The PU_5WS_5P shows lower values of 13.4 and 13.5 MJ m^−2^ compared to foams with single fillers of walnut (14.2 MJ m^−2^) and perlite (14.4 MJ m^−2^). In contrast, the PU_5WS_5V foam achieves a higher value compared to foams with single fillers, but is still lower than the reference foam. In terms of TSR, the PU_5WS_5P foam achieves a higher value of 556 m^2^ m^−2^ than the PU_10WS (537 464 m^2^ m^−2^) and PU_10P (464 537 m^2^ m^−2^), while the PU_5WS_5V foams achieve significantly lower values of 384 and of 386 m^2^ m^−2^, respectively.

In the case of carbon monoxide and carbon dioxide performance, a situation similar to that of THR values is observed, with PU_5WS_5P foams achieving lower values of both COY (0.16 kg kg^−1^) and CO_2_Y (2.42 kg kg^−1^), whereas the foam PU_5WS_5V achieves relatively high values of these parameters (0.40 and 4.38 kg kg^−1^), which fall between the results of the foams PU_10WS (0.24 and 2.79 kg kg^−1^) and PU_10V (0.44 and 4.44 kg kg^−1^). Compared to the corresponding foams with single mineral fillers, foams with walnut shells/mineral filler combinations also show reduced COY/CO_2_Y ratios of 0.07 for PU_5WS_5P and 0.09 for PU_5WS_5V, while it was 0.10 for the reference foam.

Analyzing the MARHE results, it can be observed that all the foams with filler combinations obtained lower results than the reference foam (102 kW m^−2^); in addition, they obtained results between the extreme foams with 10 parts of single fillers, reaching results lower than PU_10WS (89 kW m^−2^) foam and higher than PU_10P (71 kW m^−2^) and PU_10V (63 kW m^−2^). Compared to the reference foam, the MARHE results were reduced by approximately 24% for PU_5WS_5P and 29% for PU_5WS_5V.

When analyzing the LOI results, it can be seen that all foams with filler combinations scored lower than the reference foam (19.8%). The PU_10P foam showed the highest LOI value, indicating the best fire resistance (21.2%).

In order to compare the flammability of the foams, the total burn time (i.e., the time it took for the five UL94 test samples to burn to complete extinguishment) was determined. Based on the obtained results (Table 9), none of the samples achieved a V-0, V-1, or V2 rating. The total time of burning for all samples exceeded 100 s. This indicates that the addition of fillers in the form of walnut shells and mineral fillers still requires further research to investigate their properties in more detail.

#### 3.2.8. Thermal Conductivity

**Figure 13:**
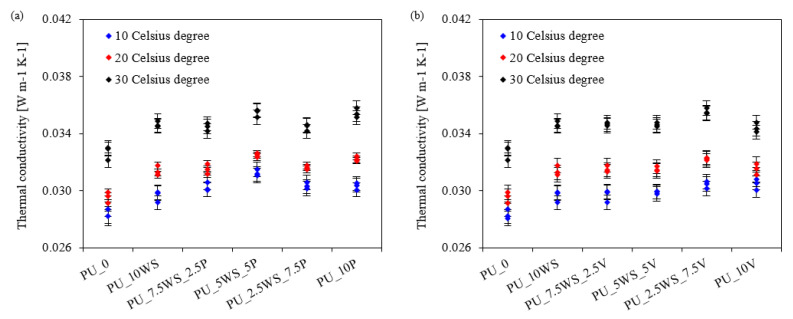
Thermal conductivity of foams with the addition of walnut shells and perlite (**a**) and walnut shells and vermiculite (**b**) at average temperatures of 10, 20, and 40 °C.

Since polyurethane foams are mainly used as thermal insulation materials, the determination of their thermal conductivity is particularly important for their application and energy efficiency. To determine the effect of natural and mineral filler combinations on the thermal conductivity of polyurethane foams, measurements were carried out at three average temperatures of 10, 20, and 40 °C, and the results are illustrated in Figure 13a,b.

When analyzing the results of polyurethane foams with the addition of walnut shells and mineral fillers, it can be observed that all foams with a combination of fillers achieved higher thermal conductivity values compared to the reference foam (10 °C–0.0281 W m^−1^ K^−1^; 20 °C–0.0296 W m^−1^ K^−1^; 40 °C–0.0329 W m^−1^ K^−1^) for all average measurement temperatures, 10, 20, and 40 °C. This is an indication of the degraded thermal insulation capabilities of modified foams. Previous work has confirmed that the increased thermal conductivity of foams containing organic and inorganic fillers is related to the presence of solid particles of the filler, which increase thermal conductivity, as well as smaller cell diameters and thicker cell walls of the foams, which affect the thermal conductivity value as well [64].

## 4. Conclusions

The presented study focused on the addition of walnut shells and mineral fillers such as perlite and vermiculite to polyurethane foams. In general, polyol systems with the addition of each part of all fillers showed increased viscosity compared to the pure system, resulting in foams with smaller cell sizes and higher density, which translated into improved mechanical properties: hardness, flexural, and compressive strength in comparison to the reference foam. Foams with added fillers also showed reduced water absorption and increased thermal conductivity compared to pure foam, and their linear dimensional changes did not exceed 1% at both reduced and elevated temperature conditions. The most important part of this section was to analyze the properties related to the flammability and thermal stability of the obtained composites since mineral fillers were used as flame retardants for polyurethane foams. Foams with added mineral fillers generally showed improved thermal stability compared to the reference foam through higher temperatures of subsequent thermal decomposition stages and higher char residue content at 600 °C. Furthermore, foams with perlite and vermiculite additions exhibited improved burning parameters, increased ignition times, reduced total heat and total smoke release, comparable to the reference foam, and lower values of COY/CO_2_Y ratio and significantly lower values of MARHE parameter than the reference foam.

## Figures and Tables

**Table 1:** Composition of polyurethane foams.

Component	Brand Name	Proportion
Polyol	Stapanpol PS-2352	100
Isocyanate	Purocyn B	160
Surfactant	Tegostab B8513	2.5
Catalysts	Kosmos 75	6
	Kosmos 33	0.8
Foaming agent	Pentane/cycliopentane	11
	Water	0.5
Nutshell	Walnut shells	0–10
Mineral filler	Vermiculite	0–10
	Perlite	0–10

**Table 2:** Composition of polyurethane foams.

	Nutshell	Amount [wt.%]	Mineral Filler	Amount [wt.%]
PU_0	-	-	-	-
PU_10WS	walnut shell	10	-	-
PUR foam with walnut shells/perlite				
PU_7.5WS_2.5P	walnut shell	7.5	perlite	2.5
PU_5WS_5P	walnut shell	5	perlite	5
PU_2.5WS_7.5P	walnut shell	2.5	perlite	7.5
PU_10P	walnut shell	-	perlite	10
PUR foam with walnut shells/vermiculite				
PU_7.5WS_2.5V	walnut shell	7.5	vermiculite	2.5
PU_5WS_5V	walnut shell	5	vermiculite	5
PU_2.5WS_7.5V	walnut shell	2.5	vermiculite	7.5
PU_2.5WS_7.5V	walnut shell	-	vermiculite	10

**Table 3:** Thermal stability results of walnut shells, vermiculite, and perlite.

Foam	1st Stage	2nd Stage	3rd Stage	Char Residue at 600 °C [wt.%]
	T_max_ [°C]			
WS	77 ± 2	296 ± 3	524 ± 3	5.2 ± 0.1
P	193 ± 2	-	-	96.4 ± 0.1
V	104 ± 2	-	-	87.8 ± 0.1

**Table 4:** Characteristic times and maximum temperature of the synthesis of polyurethane foams with the addition of walnut shells and mineral fillers.

	Start Time [s]	Expansion Time [s]	Stabilization Time [s]	Total Time [s]	Maximum Temperature [° C]
PU_0	29 ± 2	262 ± 8	351 ± 6	642 ± 6	121 ± 2
PU_10WS	29 ± 1	270 ± 7	381 ± 5	680 ± 7	129 ± 2
PU_7.5WS_2.55P	29 ± 1	273 ± 5	380 ± 9	682 ± 9	129 ± 1
PU_5WS_5P	30 ± 2	279 ± 7	379 ± 9	688 ± 9	130 ± 1
PU_2.5WS_7.5P	30 ± 2	284 ± 7	380 ± 8	694 ± 8	130 ± 1
PU_10P	32 ± 2	287 ± 6	393 ± 7	712 ± 7	131 ± 1
PU_7.5WS_2.5V	30 ± 1	277 ± 8	389 ± 7	696 ± 7	129 ± 2
PU_5WS_5V	30 ± 3	280 ± 7	405 ± 5	715 ± 6	132 ± 1
PU_2.5WS_7.5V	31 ± 2	285 ± 9	409 ± 7	725 ± 8	134 ± 2
PU_10V	31 ± 2	281 ± 6	389 ± 7	701 ± 7	134 ± 1

**Table 5:** Rheological and structural properties of polyurethane foams with the addition of walnut shells and mineral fillers.

	Dynamic Viscosity at 10 RPM [mPa·s]	Cell Diameter [µm]	Anisotropy [-]	Apparent Density [kg m^−3^]
PU_0	780 ± 10	510 ± 143	1.62 ± 0.14	35.49 ± 0.17
PU_10WS	2820 ± 10	498 ± 146	1.67 ± 0.16	38.17 ± 0.77
PU_7.5WS_2.5P	2880 ± 10	452 ± 88	1.67 ± 0.16	38.23 ± 0.34
PU_5WS_5P	3040 ± 10	431 ± 102	1.66 ± 0.24	39.89 ± 0.49
PU_2.5WS_7.5P	3270 ± 10	412 ± 111	1.67 ± 0.26	42.03 ± 0.11
PU_10P	3370 ± 10	411 ± 97	1.56 ± 0.10	43.08 ± 0.26
PU_7.5WS_2.5V	2810 ± 20	452 ± 130	1.70 ± 0.25	38.22 ± 0.08
PU_5WS_5V	2710 ± 20	450 ± 145	1.70 ± 0.29	38.79 ± 0.24
PU_2.5WS_7.5V	2750 ± 20	435 ± 130	1.68 ± 0.30	39.18 ± 0.45
PU_10V	2350 ± 20	444 ± 107	1.70 ± 0.17	39.54 ± 0.46

**Table 6:** Changes in linear dimensions of foams with the addition of walnut shells and mineral fillers, after conditioning at –20 °C and +70 °C.

	Temperature of −20 °C			Temperature of +70 °C		
Foam	Δh [%]	Δw [%]	Δt [%]	Δh [%]	Δw [%]	Δt [%]
PU_0	0.42 ± 0.02	0.73 ± 0.00	0.73 ± 0.02	0.37 ± 0.17	0.54 ± 0.19	0.65 ± 0.02
PU_10WS	0.17 ± 0.14	0.23 ± 0.18	0.26 ± 0.21	0.19 ± 0.14	0.11 ± 0.09	0.35 ± 0.21
PU_7.5WS_2.55P	0.25 ± 0.15	0.05 ± 0.02	0.04 ± 0.04	0.30 ± 0.10	0.39 ± 0.19	0.36 ± 0.04
PU_5WS_5P	0.25 ± 0.15	0.07 ± 0.02	0.18 ± 0.06	0.26 ± 0.01	0.27 ± 0.00	0.40 ± 0.06
PU_2.5WS_7.5P	0.19 ± 0.19	0.09 ± 0.06	0.05 ± 0.00	0.33 ± 0.06	0.46 ± 0.21	0.57 ± 0.00
PU_10P	0.05 ± 0.04	0.11 ± 0.01	0.25 ± 0.15	0.17 ± 0.09	0.33 ± 0.06	0.38 ± 0.04
PU_7.5WS_2.5V	0.16 ± 0.09	0.12 ± 0.05	0.08 ± 0.00	0.08 ± 0.04	0.15 ± 0.07	0.06 ± 0.00
PU_5WS_5V	0.15 ± 0.10	0.15 ± 0.02	0.14 ± 0.06	0.03 ± 0.02	0.53 ± 0.38	0.04 ± 0.06
PU_2.5WS_7.5V	0.11 ± 0.01	0.05 ± 0.05	0.06 ± 0.01	0.28 ± 0.09	0.05 ± 0.05	0.42 ± 0.01
PU_10V	0.04 ± 0.01	0.33 ± 0.04	0.18 ± 0.01	0.07 ± 0.07	0.07 ± 0.02	0.27 ± 0.07

**Table 7:** Thermal stability results of polyurethane foams with the addition of walnut shells and mineral fillers.

Foam	1st Stage	2nd Stage	3rd Stage	Char Residue at 600 °C [wt.%]
	T_max_ [°C]			
PU_0	202 ± 2	310 ± 3	585 ± 4	22.3 ± 0.1
PU_10WS	221 ± 2	319 ± 3	636 ± 3	28.9 ± 0.1
PU_7.5WS_2.5P	216 ± 3	319 ± 2	627 ± 2	32.6 ± 0.1
PU_5WS_5P	216 ± 3	319 ± 2	608 ± 4	32.7 ± 0.1
PU_2.5WS_7.5P	221 ± 2	319 ± 2	632 ± 4	33.2 ± 0.1
PU_10P	216 ± 3	319 ± 3	632 ± 3	34.2 ± 0.1
PU_7.5WS_2.5V	207 ± 2	314 ± 3	608 ± 3	30.3 ± 0.1
PU_5WS_5V	212 ± 2	319 ± 3	604 ± 3	31.6 ± 0.1
PU_2.5WS_7.5V	207 ± 3	314 ± 2	604 ± 2	31.7 ± 0.1
PU_10V	216 ± 2	314 ± 2	604 ± 4	34.0 ± 0.1

**Table 8:** Burning behavior of polyurethane foams with the addition of walnut shells and mineral fillers.

Foam	IT [s]	THR [MJ m^−2^]	TSR [m^2^ m^−2^]	COY [kg kg^−1^]	CO_2_Y [kg kg^−1^]	COY/CO_2_Y [-]	MARHE [kW m^−2^]
PU_0	4 ± 0	20.4 ± 0.8	750 ± 8	0.38 ± 0.04	3.95 ± 0.03	0.10 ± 0.01	102 ± 4
PU_10WS	6 ± 1	14.2 ± 0.6	464 ± 8	0.24 ± 0.03	2.79 ± 0.04	0.08 ± 0.01	89 ± 5
PU_5WS_5P	8 ± 1	13.4 ± 0.6	556 ± 9	0.16 ± 0.02	2.42 ± 0.04	0.07 ± 0.01	77 ± 5
PU_10P	8 ± 1	14.4 ± 0.7	537 ± 7	0.30 ± 0.02	3.69 ± 0.05	0.08 ± 0.01	71 ± 4
PU_5WS_5V	8 ± 1	19.1 ± 0.8	384 ± 6	0.40 ± 0.03	4.38 ± 0.04	0.09 ± 0.01	72 ± 4
PU_10V	8 ± 1	13.4 ± 0.8	538 ± 6	0.44 ± 0.04	4.44 ± 0.05	0.10 ± 0.01	63 ± 5

**Table 9:** The results of LOI and UL94 tests.

Foam	LOI [%]	UL-94 Test	Total Burning Time [s]
PU_0	19.8 ± 0.1	No Rating	>100
PU_10WS	21.0 ± 0.1	No Rating	>100
PU_5WS_5P	20.8 ± 0.1	No Rating	>100
PU_10P	21.2 ± 0.1	No Rating	>100
PU_5WS_5V	20.0 ± 0.1	No Rating	>100
PU_10V	20.7 ± 0.1	No Rating	>100

## Data Availability

Data are contained within the article.
